# Metabolic Scarring: The Persistent Impact of Past Obesity on Long‐Term Metabolic Health Despite Weight Loss

**DOI:** 10.1002/edm2.70086

**Published:** 2025-07-20

**Authors:** Ali Hemade, Pascale Salameh

**Affiliations:** ^1^ Faculty of Medicine Lebanese University Hadat Lebanon; ^2^ Faculty of Pharmacy Lebanese University Hadat Lebanon; ^3^ Gilbert and Rose‐Marie Chagoury School of Medicine Lebanese American University Beirut Lebanon; ^4^ Department of Primary Care and Population Health University of Nicosia Medical School Nicosia Cyprus; ^5^ Institut National de Santé Publique d'Épidémiologie Clinique et de Toxicologie‐Liban (INSPECT‐LB) Beirut Lebanon

**Keywords:** metabolic scar, NHANES, obesity, weight loss

## Abstract

**Background:**

Conventional cardiometabolic risk assessment relies primarily on a patient's current body mass index, yet individuals who have lost weight after a period of obesity may continue to harbour elevated metabolic risk. We sought to quantify the persistent impact of past obesity on glycaemic control and to develop a clinical risk score that integrates weight history with current risk factors.

**Methods:**

We performed a cross‐sectional analysis of 15,422 adults (≥ 18 years) from the 2011–2020 NHANES cycles. Participants with complete self‐reported weight history (highest adult weight, weight 1 year ago, number of ≥ 5% weight‐loss episodes) and measured BMI were included. Metabolic scarring was defined by elevated haemoglobin A1c (HbA1c ≥ 5.7%) or HOMA‐IR ≥ 2.5. We applied inverse‐probability‐weighted logistic regression to estimate the association between prior obesity and current HbA1c, adjusting for confounders. We then refit a survey‐weighted logistic model using age per decade, current BMI, weight‐history category, sex and race/ethnicity, converting regression coefficients into an integer point‐based score. Discrimination was evaluated by survey‐weighted area under the receiver‐operating characteristic curve (AUC).

**Results:**

Formerly obese individuals exhibited significantly higher HbA1c than never‐obese peers (adjusted *β* = 0.58%, *p* < 0.002), indicative of metabolic scarring. The derived risk score ranged from −31 to +90 points (median = 6; IQR = −3 to 16) and achieved an AUC of 0.79 (95% CI 0.77–0.81). Age per decade, BMI, and weight history contributed 4, 1 and up to 4 points, respectively; female sex and Non‐Hispanic White race subtracted points. Calibration across predicted‐risk deciles was excellent (slope = 0.98).

**Conclusions:**

A history of obesity imparts a lasting glycemic risk that is not captured by current BMI alone. Our metabolic scarring risk score offers a pragmatic tool for identifying individuals at elevated metabolic risk despite weight normalisation.

## Graphical abstract

Past obesity leaves a lasting metabolic footprint. Using NHANES data, we show that formerly obese individuals exhibit elevated HbA1c despite weight normalisation. We introduce a metabolic‐scarring risk score that integrates weight history to improve glycaemic risk prediction beyond current BMI.

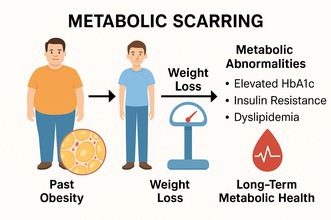

## 1 Introduction

Obesity has become a pervasive global health challenge, driving a surge in metabolic disorders. The worldwide prevalence of obesity has nearly tripled since 1975 and reached approximately 14% of adults by 2019 [1]. In the United States, over 40% of adults now meet criteria for obesity (BMI ≥ 30 kg/m^2^), up from about 30% two decades earlier [2]. Excess adiposity is closely tied to adverse health outcomes: obesity dramatically raises the risk of type 2 diabetes, cardiovascular disease, fatty liver disease and certain cancers [3]. Nevertheless, an individual's current BMI does not tell the whole story of their metabolic health status, and a growing body of evidence highlights important limitations of BMI‐based risk assessment.

One limitation is the heterogeneity of metabolic health among individuals at any given BMI. Many patients with obesity exhibit the expected metabolic derangements, but some do not—a phenotype often termed ‘metabolically healthy obesity’ (MHO) [4]. Conversely, a subset of normal‐weight individuals is metabolically unhealthy, manifesting insulin resistance or dyslipidemia despite a BMI in the normal range [4]. In one large study, obesity without metabolic abnormalities did not confer higher mortality risk compared to lean healthy individuals, whereas the presence of even a single metabolic risk factor (such as hypertension or hyperglycemia) increased mortality risk regardless of BMI [4]. BMI also fails to capture body fat distribution (visceral vs. subcutaneous) and muscle mass, further limiting its precision in risk stratification [5].

Another critical limitation of conventional BMI‐based risk assessment is the neglect of weight history. Studies have indicated that weight history could provide information about future diabetes and mortality risk above and beyond current weight [6]. For example, in the Framingham Heart Study, adults who had returned to a normal BMI after earlier obesity still had more than double the mortality rate of those who were never obese [7]. Similarly, long‐term exposure to overweight is associated with higher odds of type 2 diabetes and other complications [8]. These findings suggest that prior elevations in weight can have enduring adverse effects—a concept we define here as ‘metabolic scarring’. Metabolic scarring refers to the persistence of metabolic dysfunction or elevated disease risk that remains even after an individual has attained a normal weight. Potential examples of metabolic scarring include persistently elevated haemoglobin A1c or blood pressure in someone who formerly had obesity but is now lean. Traditional risk algorithms and screening guidelines do not account for this weight history. For instance, current U.S. recommendations focus diabetes screening on individuals with overweight or obesity in midlife [9, 10, 11], which could miss a normal‐BMI person who formerly had obesity and remains at high risk. The omission of weight history may also partly explain paradoxes in the literature, such as ‘obesity‐mortality paradox’ findings in older adults [12]. Using weight history data has been proposed as one strategy to overcome these biases, and indeed, incorporating a person's highest BMI or duration of obesity yields stronger associations with outcomes than single‐point BMI measurements [7].

Collectively, these insights point to an urgent need for improved risk assessment tools that integrate weight history. Clinicians currently lack easy methods to quantify how a patient's past weight trajectories contribute to current risk. Recent expert consensus has emphasised that collecting a weight history should become a routine part of medical care for patients with overweight or obesity [13]. Life‐course exposure to obesity—including the age of onset, maximum BMI attained and cumulative duration—all appears to impact cardiometabolic risk [13]. Considering these factors could improve risk stratification beyond the snapshot of current BMI. Metabolic scarring provides a conceptual framework for why weight history matters: prior obesity may induce lasting changes (e.g., adipocyte cell profile, inflammatory set‐points, epigenetic marks) that continue to drive disease risk even after successful weight loss. However, existing risk prediction models and guidelines have yet to incorporate this concept. There is a clear rationale to develop new tools that capture the ‘footprint’ of a patient's weight history on their metabolic health.

In light of these gaps, the present study examines metabolic scarring in an endocrinology/epidemiology context. We investigate whether individuals with a history of obesity exhibit residual metabolic dysfunction (specifically, elevated HbA1c as an indicator of glycemic risk) despite having a normal or lower BMI currently. Furthermore, we construct a novel point‐based risk score that integrates weight history, current BMI, age and other factors to improve individual risk prediction. Our goal is to determine if incorporating weight history can enhance identification of high‐risk patients (for conditions like prediabetes/diabetes) who would be overlooked by BMI‐based assessment alone. By formally introducing and quantifying ‘metabolic scarring’, we aim to lay the groundwork for more nuanced risk prediction and personalised intervention strategies for obesity‐related diseases. Ultimately, this work seeks to shift the paradigm from one‐time BMI measurements to a life‐course approach in metabolic risk assessment, with the potential to improve prevention and clinical care.

## 2 Methods

This study utilised data from the National Health and Nutrition Examination Survey (NHANES) to examine the long‐term metabolic impact of past obesity. We performed a cross‐sectional analysis incorporating advanced statistical methods to estimate the persistence of metabolic dysfunction among individuals with a history of obesity who subsequently achieved a normal body mass index (BMI).

## 3 Study Population and Data Source

We extracted data from NHANES cycles 2011–12 through 2017–20. The National Health and Nutrition Examination Survey (NHANES) is a continuous, multistage, stratified probability sample of the U.S. civilian, non‐institutionalised population conducted by the National Center for Health Statistics [2]. Trained personnel collect questionnaire, examinationand laboratory data in mobile examination centres (MECs); complex sampling weights allow national inference. We pooled five pre‐pandemic cycles (2011–2012 through 2017–March 2020) and re‐scaled 2‐year MEC weights (WTMEC2YR) to reflect the combined 10‐year sample, following NCHS analytic guidelines [2]. Inclusion criteria required participants to be at least 18 years old and have complete BMI history variables (WHQ140 [weight 1 year ago], WHQ150 [age at heaviest weight], and WHQ225 [number of intentional weight losses ≥ 5% of body weight], BMXBMI [current BMI]). Participants with a known diabetes diagnosis were excluded.

## 4 Variables and Biomarkers

Metabolic health was assessed using a panel of biomarkers, including fasting glucose (LBXGLU), HbA1c (LBXGH), insulin (LBXIN), and lipid profiles (LBDLDL, LBXTR, LBXTC). Lifetime peak adiposity was captured by the NHANES item WHQ150 (self‐reported highest adult weight), from which the highest adult BMI (peak‐BMI) was calculated. Current weight 1 year ago (WHQ140) was used solely to derive a ‘recent weight‐change’ covariate (kg·year^−1^) and was not part of our primary weight‐history classification. Participants were categorised as: (i) Always Normal (peak‐BMI < 25 kg·m^−2^), (ii) Formerly Overweight (25 ≤ peak‐BMI < 30 and current BMI < 25), (iii) Formerly Obese (peak‐BMI ≥ 30 and current BMI < 30), (iv) Still Overweight and (v) Still Obese. Demographic covariates included age, sex, race, socioeconomic status (income‐to‐poverty ratio) and education level. HOMA‐IR [14] was calculated using the formula:

$$\text{HOMA}-\mathrm{IR}=\frac{\text{fasting insulin}\;\left[\mu \frac{U}{\mathrm{mL}}\right]\times \text{fasting glucose}\left[\mathrm{mg}/\mathrm{dL}\right]}{405}$$

## 5 Statistical Analysis

Variable‐level missingness is summarised in Table S1. Laboratory items collected in fasting subsamples (fasting glucose, insulin and lipids) showed expected structural missingness (≈45%). Little's MCAR test was significant (*p* < 0.001) which indicated that data were missing at random (MAR). The primary analyses therefore used complete‐case, survey‐weighted inverse‐probability models; to evaluate robustness we generated 20 multiply‐imputed data sets via chained equations (mice v3.16) incorporating all analysis variables plus predictors of missingness (age, sex, race/ethnicity, BMI, examination session). Pooled estimates are presented in Table S2.

Exploratory data analysis was conducted to evaluate variable distributions and detect potential outliers. Weighted means and standard errors (SE) were calculated for continuous biomarkers, while weighted proportions were derived for categorical variables using NHANES survey weights to ensure population representativeness.

IPW regression models were applied to estimate the association between past obesity and metabolic outcomes while adjusting for full weight history variables. We applied NHANES MEC sampling weights (WTMEC2YR), strata (SDMVSTRA) and cluster (SDMVPSU) in all IPW models.

To translate our multivariable model into a practical clinical tool, we derived a pointbased ‘metabolic scarring’ risk score as follows. Metabolic scarring was defined as the presence of elevated HbA_1_c (≥ 5.7%), coded as a binary outcome variable. We chose HbA_1_c ≥ 5.7% as the primary indicator of metabolic scarring because HbA_1_c integrates glycemic exposure over ~120 days and predicts incident diabetes independent of transient glucose excursions [15]. Moreover, erythrocyte protein glycation embodies the concept of a lasting ‘metabolic memory’ [16]. Sensitivity analyses repeated the primary models with (a) fasting plasma glucose (≥ 100 mg·dL^−1^‐) and (b) homeostasis‐model insulin resistance (HOMA‐IR ≥ 2.5) as alternate outcomes. We first refit the survey‐weighted logistic regression using age expressed per decade (RIDAGEYR/10) alongside BMI, a simplified five‐level weight‐history variable (Always Normal; Formerly Overweight; Formerly Obese; Still Overweight; Still Obese; with rare categories collapsed into ‘Other Status’), sex, and a three‐level race/ethnicity variable (NonHispanic White; NonHispanic Black; Other). Regression coefficients were then multiplied by 10 and rounded to the nearest integer to assign each predictor a point value. An individual's total score is calculated by summing the points corresponding to their age, BMI, weight‐status group, sex and race; this sum is converted back to a predicted probability of metabolic scarring via the inverse‐logit transformation (plogis‐[score/10]). We assessed discrimination by the survey‐weighted area under the ROC curve and calibration across deciles of predicted risk. An interactive Shiny dashboard implementing this scoring algorithm—allowing clinicians to input patient values, view real‐time risk estimates, and inspect validation plots—is publicly available.

In our weight‐cycling analysis, we derived a continuous weight‐cycling intensity score by standardising the number and magnitude of self‐reported ≥ 10 lbs. intentional weight‐loss episodes (WHQ225) across the analytic sample. We then examined the dose–response relationship between this standardised' weight‐cycling' core and each metabolic outcome (HbA1c, HOMA‐IR, LDL cholesterol, and triglycerides) using survey‐weighted multivariable polynomial regression. For each outcome, we specified a series of orthogonal polynomial terms—linear, quadratic, cubic and quartic—for the weight‐cycling score to allow for flexible, non‐monotonic dose–response shapes. Models were adjusted for age, sex, current BMI and self‐reported race/ethnicity. To make the dose–response findings more clinically interpretable, we collapsed the standardised weight‐cycling z‐scores into tertiles, corresponding to low (<−0.67 SD), moderate (−0.67 to +0.67 SD), and high (> + 0.67 SD) levels of weight cycling. In our sample, these cut‐points approximate 0–1, 2–3 and ≥ 4 lifetime episodes of intentional ≥ 10 lbs. weight loss and regain, respectively. We then computed the predicted change in each metabolic marker by plugging the mean *z*‐score within each tertile into the fitted polynomial model.

All *p* values across metabolic outcomes were adjusted using the Benjamini–Hochberg false discovery rate at 5%.

## 6 Ethical Considerations

NHANES is a publicly available, de‐identified dataset; all participants provided informed consent. Our secondary analysis of de‐identified data was exempt from further institutional review.

## 7 Results

### 7.1 Sample Characteristics

A total of 15,422 participants met the inclusion criteria. Figure S1 presents the analytic flow diagram: 49694 participants were part of the full cohort for the selected years; after sequential exclusions for age < 18 years (*n* = 10,212), pregnancy (*n* = 476), missing weight‐history variables (*n* = 20,534) or key covariates (*n* = 3050), the final analytic sample comprised 15,422 participants (weighted *N*≈146 million). The weighted mean BMI for the study population was 29.49 (SE: 0.15). The mean HbA1c level was 5.66% (SE: 0.018), while mean fasting insulin was 13.81 μU/mL (SE: 0.25). Mean triglycerides were 110.99 mg/dL (SE: 1.15) (Table 1).

**TABLE 1:** Weighted means of key biomarkers.

Biomarker	Mean	Standard error (SE)
Body Mass Index (BMI)	29.49250	0.1487
Glycohemoglobin (%)	5.66173	0.0181
Insulin (μU/mL)	13.80906	0.2460
LDL cholesterol (mg/dL)	112.35204	0.5491
Triglycerides (mg/dL)	110.99639	1.1502

### 7.2 IPW Regression Results

IPW analysis is presented in Table 2. Compared to individuals who were never overweight, formerly overweight individuals had significantly higher HbA1c levels (*β* = 0.567, *p* = 0.0018), while those who remained obese had the highest increase (*β* = 1.039, *p* = 0.0007). Insulin resistance, measured by HOMA‐IR, was also higher in still obese individuals (*β* = 5.127, *p* = 0.0770). No significant associations were observed between weight history and LDL cholesterol or triglycerides.

**TABLE 2:** IPW‐adjusted regression results: association between weight history and metabolic markers.

Metabolic outcome	Weight status	Estimate (*β*)	Standard error	*p*
HbA1c	Formerly overweight	**0.567**	0.163	**0.0018**
	Formerly obese	0.397	0.285	0.1751
	Still overweight	0.550	0.291	0.0693
	Still obese	**1.039**	0.273	**0.0007**
	Reduced to overweight	0.604	0.299	0.0541
HOMA‐IR	Formerly overweight	−1.228	2.027	0.5498
	Formerly obese	0.303	2.296	0.8962
	Still overweight	2.044	2.700	0.4560
	Still obese	**5.127**	2.785	**0.0770**
LDL cholesterol	Formerly overweight	−7.214	5.476	0.1992
	Formerly obese	−7.114	12.55	0.5757
	Still overweight	8.406	10.04	0.4099
	Still obese	1.019	7.278	0.8898
Triglycerides	Formerly overweight	2.839	10.72	0.7933
	Formerly obese	31.67	18.65	0.1014
	Still overweight	38.91	24.25	0.1207
	Still obese	16.52	10.90	0.1419
WBC	Formerly overweight	0.709	0.639	0.2775
	Formerly obese	−0.575	0.922	0.5388
	Still overweight	0.328	0.820	0.6924
	Still obese	0.582	0.717	0.4244

*Note:* Reference Group: Always normal BMI, adjusted for age, sex and ethnicity using inverse probability weighting. Values in bold are statistically significant (*p* < 0.05).

Table 3 presents the final survey weighted logistic regression coefficients and their corresponding integer point values for the metabolic scarring score. The intercept (*β* = −4.88) was scaled to −49 points; age per decade (*β* = 0.37) contributes +4 points; each 1 kg/m^2^ of BMI (*β* = 0.13) adds +1 point; formerly obese and still obese each add +4 points, formerly overweight subtracts 2 points, still overweight adds +1 point, and all other weight history statuses contribute 0 points; female sex subtracts 2 points; Non Hispanic White subtracts 8 points; Non Hispanic Black subtracts 1 point; and Other race subtracts 1 point.

**TABLE 3:** Coefficients and point values for the metabolic scarring risk score.

Term	*β* (coef)	Points
(Intercept)	−4.88	−49
Age (per 10 years)	0.37	4
BMI (per 1 kg/m^2^)	0.13	1
Weight status—formerly obese (vs. normal BMI)	0.44	4
Weight status—formerly overweight (vs. normal BMI)	−0.17	−2
Weight status–still obese (vs. normal BMI)	0.42	4
Weight status–still overweight (vs. normal BMI)	0.10	1
Weight status–other^a^ (vs. normal BMI)	0.02	0
Gender–female (vs. male)	−0.22	−2
Race–NonHispanic Black (vs. Hispanic)	−0.11	−1
Race2–NonHispanic White (vs. Hispanic)	−0.80	−8
Race2–other^b^ (vs. Hispanic)	−0.14	−1

^a^ ‘Other’ in the Race variable encompasses all self‐identified race/ethnicity groups beyond Hispanic, Non‐Hispanic White and Non‐Hispanic Black as defined by NHANES (including Asian, Native American and multiracial respondents), collapsed for analytic stability.

^b^ ‘Other’ in the weight status variable comprises participants whose self‐reported weight‐history patterns did not meet the predefined criteria for the five primary categories (always normal; formerly overweight; formerly obese; still overweight; still obese)—for example, those with modest (< 5%) weight fluctuations, ambiguous or incomplete recall of peak weight or rare sequences of weight loss and regain that could not be assigned to a main group.

**FIGURE 1:**
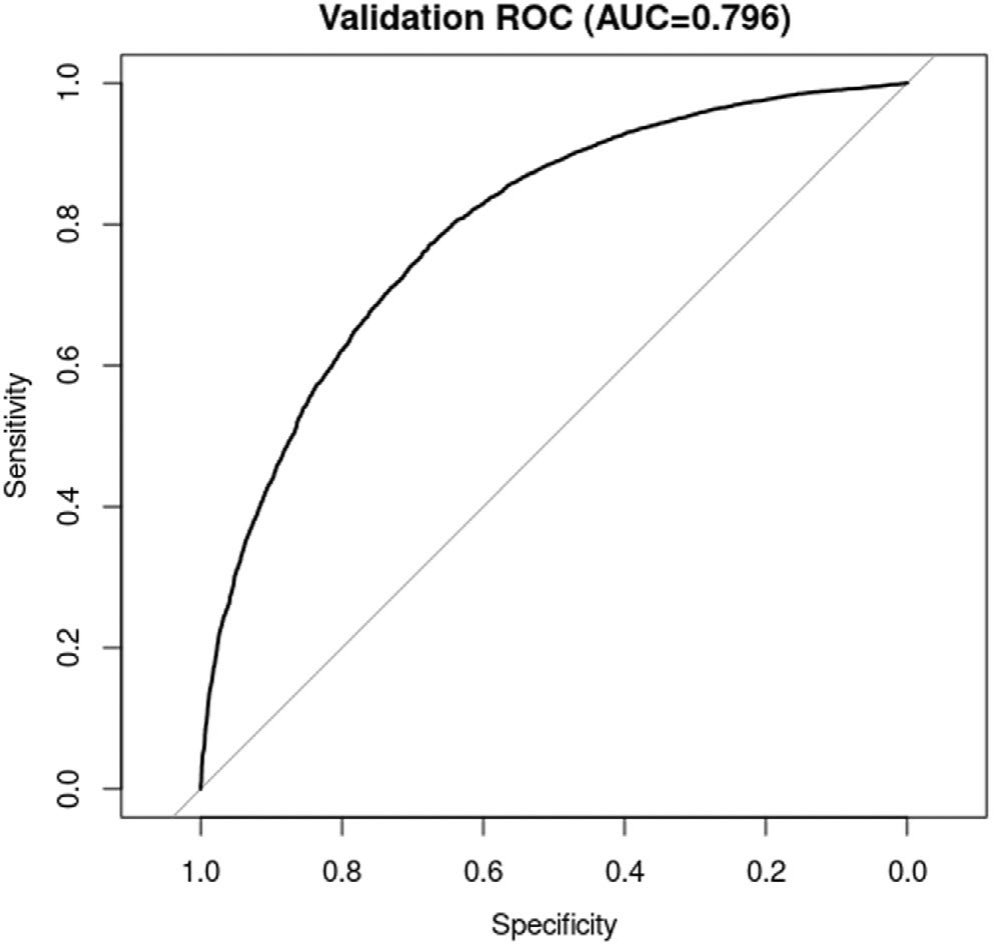
Receiver‐operating characteristic curve for metabolic scarring risk score in the validation cohort (AUC = 0.796).

When applied to our analytic sample (*n* = 15,422), total scores ranged from −31 to 90 (median = 6, interquartile range = −3 to 16). Discrimination was good, with a surveyweighted area under the ROC curve of 0.79 (95% CI 0.77–0.81), as shown in Figure 1.

**FIGURE 2:**
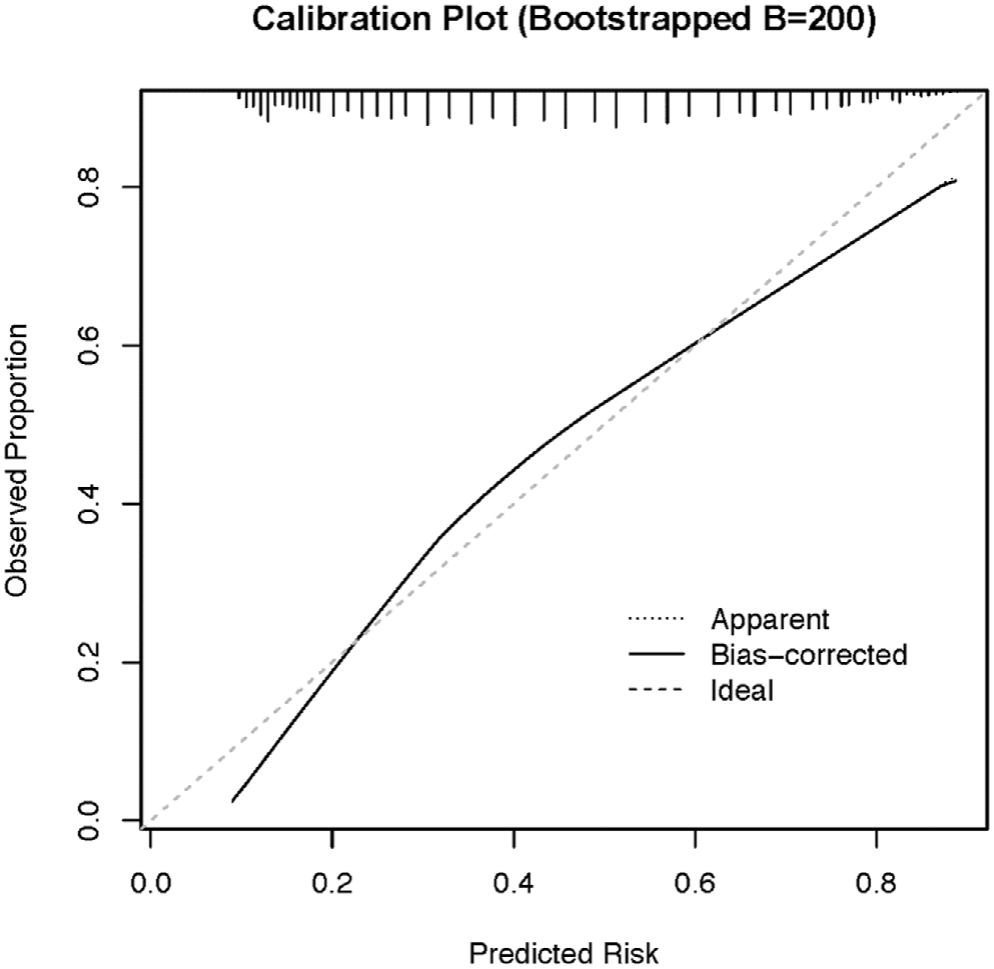
Calibration plot of the metabolic scarring score.

**FIGURE 3:**
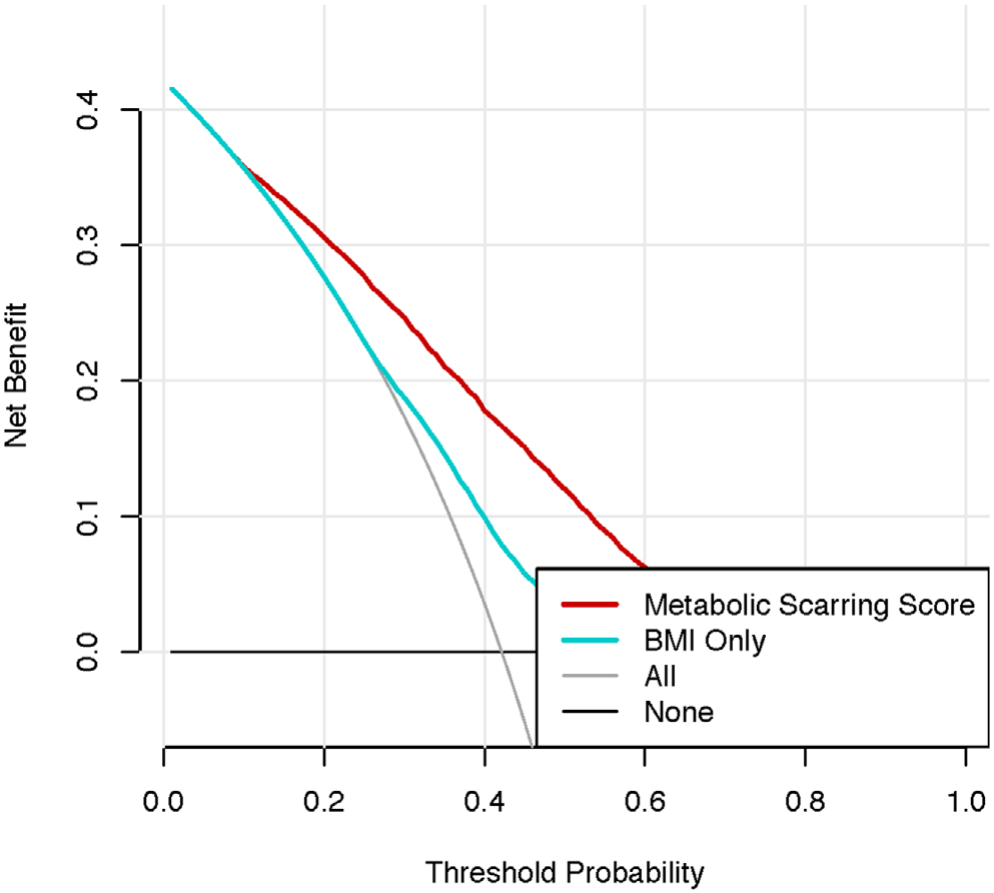
Decision curve analysis comparing full score versus BMI‐only model. For instance, a 35 year‐old (3.5 decades × 4 points = 14) with BMI 27 kg/m^2^ (× 1 point = 27), always normal weight history (0 points), female (−2 points), and Non Hispanic Black (−1 point) yields a total of 14 + 27 − 2 − 1 − 49 = −11 points, corresponding to a predicted metabolic scarring risk of‐ plogis(−11/10) ≈ 25%. An interactive Shiny application implementing this scoring algorithm—complete with realtime score calculation, ROC curve and calibration plot—is publicly available at https://alihemade.shinyapps.io/ObesityMet/.

To assess the incremental value of lifetime weight history, we compared three nested logistic regression models for metabolic scarring (Table 4). The base model including BMI alone yielded an AUC of 0.638, McFadden *R* ^2^ of 0.037, and BIC of 20,234.82. Adding weight history improved discrimination (AUC = 0.643), fit (*R* ^2^ = 0.048) and reduced BIC to 20,059.94. The full model incorporating age, sex, race/ethnicity, BMI and weight history achieved substantially better performance, with an AUC of 0.795, McFadden *R* ^2^ of 0.207 and BIC of 16,771.15 (Figures 2 and 3).

**TABLE 4:** Model comparison showing improvement in predictive performance with the addition of weight history and demographic covariates.

Model	AUC	McFadden *R* ^2^	BIC
BMI only	0.638	0.037	20,234.82
+ Weight history	0.643	0.048	20,059.94
Full model (score)	0.795	0.207	16,771.15

Using fasting glucose or HOMA‐IR as alternate outcomes yielded similar patterns (Table S3). The metabolic‐scarring score retained strong discrimination (AUC = 0.78 and 0.80, respectively).

## 8 Discussion

In this study, we provide evidence that a history of obesity can leave lingering metabolic effects—a phenomenon of ‘metabolic scarring’. Our key findings were two‐fold. First, even after substantial weight loss, individuals with prior obesity showed persistently higher HbA1c levels (and related glycemic risk) compared to never‐obese peers of the same current BMI. Second, we translated this insight into a practical risk prediction tool. We developed a point‐based risk score that incorporates age, current BMI and weight history (particularly the peak lifetime BMI and duration of obesity) to estimate an individual's risk of dysglycemia. The proposed score demonstrated that considering weight history alongside current adiposity markedly improves risk stratification for abnormal HbA1c.

Our results align with and extend prior research in several important ways. The persistent elevation in HbA1c among those with former obesity is consistent with earlier observations that weight loss, while beneficial, often does not completely normalise risk profiles. For example, a recent analysis of > 500,000 individuals in a UK database found that a person who lost weight from a BMI of ~35 to ~30 still had about 11% higher risk of developing type 2 diabetes than a never‐obese individual at BMI 30 [17]. Similarly, that study noted residual risk for sleep apnea and osteoarthritis after weight loss—that is, some risk remained elevated compared to persons who were always at the lower weight [17]. Our findings confirm in a glycemic context that prior obesity confers a lasting excess risk, even after accounting for current body size. The concept of metabolic scarring offers an explanation for why the ‘formerly obese’ do not revert to the same low‐risk state as the ‘never obese’. It also dovetails with literature on weight cycling: repeated cycles of weight loss and regain have been linked to higher incidence of type 2 diabetes [18] and greater mortality risk [8, 19, 20], suggesting that the body ‘remembers’ past obesity in a metabolically deleterious way. By formally introducing a metabolic scarring construct, our study extends these observations and provides a quantitative framework to capture risk associated with weight history.

Crucially, we demonstrated that incorporating weight history can improve individual risk prediction. Current clinical practice predominantly uses cross‐sectional metrics—for instance, screening guidelines target people above a BMI threshold at a certain age [11]. Such approaches may fail to identify a patient who has transitioned from obesity to normal weight but still carries elevated risk. Our new point‐based risk score addresses this gap by assigning additional points for a history of high BMI or longer obesity duration. This means that an older adult of normal BMI who once had severe obesity would receive a high‐risk score, flagging them for proactive screening or interventions. From a clinical perspective, this tool could enable more personalised risk assessment. For example, two individuals with BMI 25 kg/m^2^ in clinic today might have very different weight histories—one always lean, one formerly obese—and our model predicts the latter is at significantly higher risk for diabetes. In practice, that could translate to earlier glucose testing, lifestyle counselling, or even preventive pharmacotherapy for the high‐score patient, whereas the other might be managed with routine monitoring. The addition of weight history thus refines risk stratification beyond current BMI and age alone. This corroborates calls from experts to incorporate weight trajectories into patient evaluations [13].

The notion that obesity leaves lasting biological ‘scars’ is biologically plausible and supported by emerging mechanistic evidence. One potential mechanism is chronic inflammation. Adipose tissue from formerly obese individuals does not simply revert to a completely lean state; studies in murine models indicate that obesity induces an imprinted immune profile in fat that endures through weight loss and can even worsen with weight regain [21]. Obesity‐conditioned macrophages and T cells in adipose tissue remain in a pro‐inflammatory, insulin‐resistant state (sometimes termed ‘trained immunity’ or innate immune memory) that can reactivate quickly if weight is regained [21, 22]. Such a persistent inflammatory milieu could contribute to residual insulin resistance, explaining elevated HbA1c despite currently normal weight. Another mechanism is adipocyte cell biology. Severe obesity increases the number of adipocytes, as the body recruits new fat cells (hyperplasia) in addition to expanding existing ones (hypertrophy); that is, when an obese person loses weight, the size of fat cells shrinks but the number of adipocytes does not fully decrease [23]. They are left with many small adipocytes. These residual cells may be pathologically primed—for instance, having altered adipokine secretion or reduced capacity to store lipid safely, and the persistence of an expanded adipocyte pool could predispose to rapid fat redistribution to visceral depots or ectopic sites with even modest weight regain, thereby maintaining a higher baseline cardiometabolic risk [24]. There is compelling evidence that prior nutritional and metabolic exposures can leave stable epigenetic marks on genes regulating metabolism. A recent study demonstrated that human adipose tissue retains transcriptional and epigenomic changes long after weight reduction—effectively an ‘obesogenic memory’ encoded in the cells [24]. In mice, these obesity‐induced epigenetic modifications (such as persistent histone methylation at inflammatory gene loci) led to accelerated weight regain and blunted insulin sensitivity upon return to a high‐fat diet [24]. Such molecular scars could underlie the difficulty many patients experience in maintaining weight loss and the residual risk we observe. While direct mechanistic measurements were beyond our study's scope, the convergence of epidemiologic and molecular evidence strongly suggests that past obesity leaves a pathophysiological legacy—through inflammation, adipocyte dysfunction, and epigenetic reprogramming—that manifests as persistent metabolic risk. Our concept of metabolic scarring offers a unifying framework to study these phenomena. Future research integrating clinical data with molecular profiling (e.g., examining epigenetic markers or inflammatory profiles in formerly obese individuals with normal BMI) would be valuable to pinpoint the exact biological mediators of metabolic scarring.

While this study advances our understanding of weight history and risk, it has important limitations. NHANES provides only cross‐sectional snapshots of lifetime peak weight; the precise timing and duration of peak adiposity could not be reconstructed. Longitudinal electronic‐health‐record cohorts with serial weights would allow finer temporal modelling of scarring.

Second, weight history in our study was obtained by self‐report, introducing potential recall bias. Any random error in recall would likely bias toward underestimating the true impact of weight history. Third, we relied on a single measurement of HbA1c to classify metabolic status. HbA1c, while a robust marker, can be influenced by haemoglobin variants or other factors, and a one‐time lab may not capture transient fluctuations. That said, HbA1c reflects roughly 3‐month average glucose, so it is a more stable indicator than fasting glucose. Fourth, our risk score, while internally derived, requires external validation. We derived point values based on associations in our dataset; these need calibration and testing in independent populations to ensure generalisability. Additionally, the score currently focuses on glycaemic risk (elevated HbA1c) as the outcome; its utility for predicting other outcomes (e.g., incident diabetes, cardiovascular events) remains to be tested. Finally, residual confounding cannot be fully eliminated. Although we adjusted for major factors, there may be unmeasured variables differentiating those who succeed in weight loss from those who do not—for instance, health consciousness, diet quality or genetic predispositions—that also affect HbA1c.

Looking ahead, our study opens several avenues for future research. Prospective studies are needed to confirm that our weight‐history‐based risk score predicts outcomes better than traditional models. Another priority is mechanistic investigation: now that we see a clear clinical signal of metabolic scarring, what are the underlying biological drivers? Tissues like liver, muscle, pancreas and adipose could be studied in weight‐loss cohorts to identify persistent changes (in gene expression, epigenetic marks, cell composition) associated with prior obesity. From a clinical trial standpoint, an intriguing next step would be to evaluate the utility of our risk tool in practice. One could envision a trial where clinicians use the metabolic scarring risk score to guide screening or prevention, and compare outcomes to standard care.

In summary, obesity leaves a durable metabolic footprint that outlasts weight normalisation. Our point‐based score operationalises this concept and improves dysglycaemia risk stratification beyond current BMI alone. Future work should prospectively validate the score in longitudinal cohorts, elucidate epigenomic and immunologic mechanisms of metabolic scarring, and test whether score‐directed screening or preventive pharmacotherapy reduces diabetes incidence.

## Author Contributions

Ali Hemade conceived the study, performed data extraction and statistical analyses, and drafted the manuscript. Pascale Salameh assisted with critical revision of the manuscript. All authors read and approved the final manuscript.

## Ethics Statement

This study used deidentified data from the publicly available NHANES database and did not involve direct patient contact or the use of individually identifiable health information. Under the U.S. Common Rule, research using only publicly available, deidentified data is exempt from institutional review board oversight; therefore, ethics approval and patient consent were not required.

## Consent

The authors have nothing to report.

## Conflicts of Interest

The authors declare no conflicts of interest.

## Supporting information


**Figure S1.** Study participant flow diagram. Sequential exclusions applied to construct the final analytic sample. Of 27,085 NHANES participants with laboratory and examination data, we excluded individuals with missing weight history or key covariates (*n* = 4062), those younger than 18 years (*n* = 4090), pregnant participants (*n* = 508) and those with known diabetes diagnoses (*n* = 3003). The final analytic sample included 15,422 adults with complete data and no prior diagnosis of diabetes.

**Table S1.**Variable‐level missingness.

**Table S2.**Multiple‐imputation versus complete‐case IPW models (HbA1c ≥ 5.7%).

**Table S3.**Sensitivity outcomes.

## Data Availability

The dataset analyzed during the current study is available in the NHANES repository: https://www.cdc.gov/nchs/nhanes/.
