# Supplementary material for: Metabolic Scarring: The Persistent Impact of Past Obesity on Long‐Term Metabolic Health Despite Weight Loss
Source: Endocrinol Diabetes Metab. 2025 Jul 20;8(4):e70086. doi: 10.1002/edm2.70086 (PMC12276455; doi:10.1002/edm2.70086)
Supplement: Supplementary file 1 — Figure S1. Study participant flow diagram. Sequential exclusions applied to construct the final analytic sample. Of 27,085 NHANES participants with laboratory and examination data, we excluded individuals with missing weight history or key covariates (n = 4062), those younger than 18 years (n = 4090), pregnant participants (n = 508) and those with known diabetes diagnoses (n = 3003). The final analytic sample included 15,422 adults with complete data and no prior diagnosis of diabetes. [file EDM2-8-e70086-s002.docx]

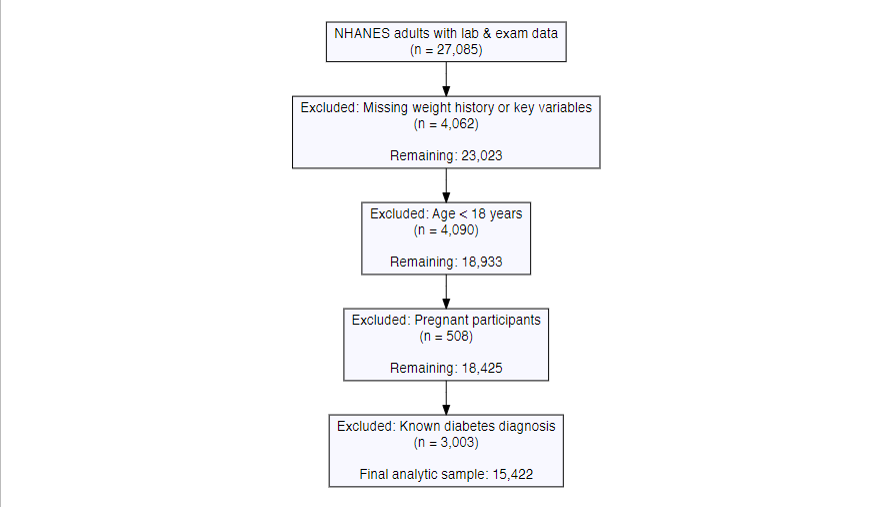


**Figure S1. Study Participant Flow Diagram**

*Sequential exclusions applied to construct the final analytic sample. Of 27,085 NHANES participants with laboratory and examination data, we excluded individuals with missing weight history or key covariates (n = 4,062), those younger than 18 years (n = 4,090), pregnant participants (n = 508), and those with known diabetes diagnoses (n = 3,003). The final analytic sample included 15,422 adults with complete data and no prior diagnosis of diabetes.*
