# Supplementary material for: Metabolic Scarring: The Persistent Impact of Past Obesity on Long‐Term Metabolic Health Despite Weight Loss
Source: Endocrinol Diabetes Metab. 2025 Jul 20;8(4):e70086. doi: 10.1002/edm2.70086 (PMC12276455; doi:10.1002/edm2.70086)
Supplement: Supplementary file 2 — Table S1.Variable‐level missingness. [file EDM2-8-e70086-s004.docx]

**Table S1 – Variable-level missingness**

| Variable | % Missing |
| --- | --- |
| SEQN | 0.00 |
| SDMVPSU | 0.00 |
| SDMVSTRA | 0.00 |
| WTINT2YR | 0.00 |
| WTMEC2YR | 0.00 |
| RIDAGEYR | 0.00 |
| RIAGENDR | 0.00 |
| RIDRETH1 | 0.00 |
| INDFMPIR | 10.49 |
| WHD140 | 13.25 |
| WHQ150 | 13.25 |
| WHQ225 | 11.60 |
| BMXBMI | 12.57 |
| LBXTR | 14.88 |
| LBDLDL | 10.23 |
| LBXWBCSI | 18.63 |
| LBXIN | 15.23 |
| LBXGL | 11.26 |
