# Supplementary material for: Metabolic Scarring: The Persistent Impact of Past Obesity on Long‐Term Metabolic Health Despite Weight Loss
Source: Endocrinol Diabetes Metab. 2025 Jul 20;8(4):e70086. doi: 10.1002/edm2.70086 (PMC12276455; doi:10.1002/edm2.70086)
Supplement: Supplementary file 3 — Table S2.Multiple‐imputation versus complete‐case IPW models (HbA1c ≥ 5.7%). [file EDM2-8-e70086-s003.docx]

**Table S2 – Multiple-imputation vs complete-case IPW models (HbA1c ≥ 5.7 %)**

| Weight-history group | Complete-case aOR (95 % CI) | Imputed aOR (95 % CI) |
| --- | --- | --- |
| Formerly Overweight | 1.38 (1.22–1.55) | 1.35 (1.19–1.52) |
| Formerly Obese | 1.68 (1.42–1.98) | 1.68 (1.42–1.98) |
| Still Overweight | 1.51 (1.30–1.75) | 1.49 (1.28–1.73) |
| Still Obese | 2.12 (1.76–2.55) | 2.10 (1.75–2.52) |

Reference = Always Normal; models adjusted for age, sex, race/ethnicity, BMI, survey design.
