# Supplementary material for: Metabolic Scarring: The Persistent Impact of Past Obesity on Long‐Term Metabolic Health Despite Weight Loss
Source: Endocrinol Diabetes Metab. 2025 Jul 20;8(4):e70086. doi: 10.1002/edm2.70086 (PMC12276455; doi:10.1002/edm2.70086)
Supplement: Supplementary file 4 — Table S3.Sensitivity outcomes. [file EDM2-8-e70086-s001.docx]

**Table S3 – Sensitivity outcomes**

| Outcome | Metric | Formerly Obese vs Always Normal aOR (95 % CI) | Score AUC |
| --- | --- | --- | --- |
| Fasting glucose ≥ 100 mg·dL⁻¹ | IPW logistic | 1.59 (1.31–1.92) | 0.78 |
| HOMA-IR ≥ 2.5 | IPW logistic | 1.62 (1.35–1.95) | 0.80 |
